# Pet Ownership and the Risk of Arterial Hypertension and Cardiovascular Disease

**DOI:** 10.1007/s11906-022-01191-8

**Published:** 2022-04-22

**Authors:** Stanisław Surma, Suzanne Oparil, Krzysztof Narkiewicz

**Affiliations:** 1 Faculty of Medical Sciences in Katowice, Medical University of Silesia, Katowice, Poland; 2 Club of Young Hypertensiologists, Polish Society of Hypertension, Gdańsk, Poland; 3 Department of Medicine, School of Medicine, University of Alabama at Brimingham, Brimingham, AL USA; 4 Chair and Department of Hypertension and Diabetology, Medical University of Gdańsk, Dębinki 7, 80-952 Gdańsk, Poland

**Keywords:** Home pets, Pet ownership, Blood pressure, Arterial hypertension, Cardiovascular disease

## Abstract

**Purpose of Review:**

Hypertension prevention and cardiovascular risk reduction are cornerstones in the prevention and treatment of cardiovascular diseases. Potential applicability of nontraditional cardiovascular risk reduction methods, such as pet ownership, raises a growing interest.

**Recent Findings:**

Studies show that having pets may reduce the risk of death from any cause, particularly from cardiovascular causes. Furthermore, results of some studies indicate that having pets may reduce the risk of developing hypertension and improve blood pressure control in patients with established hypertension. In addition, there is evidence that having pets may improve the prognosis of patients after myocardial infarction and stroke. One of the most important cardioprotective mechanisms of pet ownership is reduction in activity of the sympathetic nervous system.

**Summary:**

Pet ownership has a positive effect on the cardiovascular system, likely related to antihypertensive and cardioprotective mechanisms.

## Introduction

Pharmacotherapy and lifestyle modifications are the cornerstones in the prevention and treatment of cardiovascular diseases (CVDs). The potential applicability of nontraditional CVD risk reduction methods, such as pet ownership, has become an area of growing interest in recent years. Having a pet, especially a dog or a cat, has become increasingly popular worldwide. In 2016, 48 million American households had 77 million dogs, 28% of which were rescued from animal shelters [1]. In Poland, according to a survey conducted by Polish Public Opinion Research Center (TNS OBOP) in 2018, 48% of people owned a pet. The aim of this review is to summarize current evidence regarding the link between pet ownership, hypertension, and CVD risk.

## Pet Ownership and Mortality Risk in the General Population and in Patients with CVD

The relationship between pet ownership and mortality risk has been a subject of research since the 1980s. In their recently published meta-analysis of 10 studies with over 3.8 million participants, Kramer et al. showed that ownership of a dog was associated with a 24% reduction in risk of all-cause mortality (relative risk; RR = 0.76; 95% *CI*: 0.67–0.86) [2••]. An even greater (up to 65%) reduction in risk of all-cause mortality was seen in patients with previous coronary events who lived in a house with dogs (RR = 0.35; 95% *CI*: 0.17–0.69). In a subanalysis of studies with a follow-up of > 10 years, the beneficial effect of having a dog on the risk of all-cause mortality was sustained over time (RR = 0.73; 95% *CI*: 0.64–0.84). In particular, people who had a dog had a significantly reduced risk of death from CVD (RR = 0.69; 95% *CI*: 0.67–0.71) [2••].

These results were not fully confirmed in the meta-analysis published in the same year by Yeh et al. [3••]. The meta-analysis included 12 studies with 488,986 participants and a mean follow-up time of 8.7 ± 6.3 years. It showed that having an animal (cat, dog) was not clearly related to risk of death from CVD cause [significant reduction of 7% (95% *CI*: 0.86–0.99) only in healthy persons] or risk of CVD [(significant reduction of 29% (95% *CI*: 0.60–0.84) only in persons with established CVD]. Pet ownership did not influence the risk of all-cause mortality in this study [3••]. In a meta-analysis published in 2020 by Bauman et al., the results of the meta-analysis by Kramer et al. were revised by adjusting for many risk factors that could modify the effects of having a dog. Having a dog significantly reduced the risk of death from any cause by 61% only in people with previous CVD (heart rate (HR) = 0.39; 95% *CI*: 0.20–0.77) [4••]. The latest meta-analysis of 26 studies by El-Qushayri et al., which took into account multiple risk factors, showed that people who had a home pet had a 19% lower risk of death from CVD (HR = 0.81; 95% *CI*: 0.68–0.97). A subgroup analysis found that the greatest reduction in the risk of death from CVD occurred in those who owned a cat (HR = 0.79; 95% *CI*: 0.63–0.99) [5••].

Overall, having a pet was associated with lowering of CVD risk in patients with established CVD but not in healthy subjects [2••, 3••, 4••, 5••]. A summary of the above meta-analyzes is presented in Table 1.

Based on the results of the above meta-analyses, it is not possible to conclusively determine whether owning pets is associated with a lower risk of all-cause mortality, but owning pets can reduce the risk of death from CVD causes. Moreover, having pets may reduce risk of CVD events in patients with established CVD [2••, 3••, 4••, 5••].

## Pet Ownership and the Risk of Hypertension

The effect of pet ownership on blood pressure (BP) is controversial. Results of older observational studies analyzing the link between pet ownership and hypertension are inconclusive [6–8]. After adjusting for age and other confounders, pet ownership was not associated with BP increases or risk of hypertension. Furthermore, a recent National Health and Nutrition Examination Survey demonstrated that even after considering potential confounding factors, having a pet (cat or dog) was an independent predictor of lower hypertension risk (Table 2) [9••]. However, a meta-analysis of 11 studies found that persons with a pet had a 1.7 mmHg (95% *CI*: − 3.062 to − 0.310 mmHg) lower systolic BP (SBP), a non-significant reduction in diastolic BP (DBP) [mean diastolic BP = − 0.23 (95% *CI*: − 1.05 to 0.60)], and a significant decrease in HR of 2.3 bpm (95% *CI*: − 3.074 to − 1.573 bpm) [5••]. The BP lowering effect was especially evident during direct contact with the pet [10], and influenced by the intesity of the owner-pet relationship [11], species of the pet [9••, 12] and breed of dog [13].

A study by Xu et al. assessed the effect of having pets on BP in 9354 children and adolescents aged 5–17 years in China [14•]. Over 1/5 of the participants had current exposures to pets and 10.6% of participants had dogs. Children who had a dog had a significantly lower (32–34%) risk of hypertension. Furthermore, prior exposure to a pet during fetal life significantly reduced the risk of hypertension by 34% (95% *CI*: 0.45–0.97) in men [14•]. Another study by Xu et al. assessed the effect of having pets on the risk of developing hypertension in children who were exposed toenvironmental tobacco smoke (ETS) [15•]. Those who were not exposed to pets were at increased risk of developing hypertension compared to those exposed to pets, and the protective effect of pet ownership increased with a greater number of pets in the home. Exposure to in utero ETS was associated with hypertension (aOR) = 1.32; 95% *CI*: 1.13–1.54) only for those children without pet exposure in utero but not for those with pets (aOR = 0.75; 95% *CI*: 0.49–1.15). Household dog ownership was associated with significantly lower effects of ETS on hypertension and the associations between ETS and pet ownership were more robust for girls than for boys and for younger than older children [15•]. A study by Lawrence et al., which included the same group of children as the studies by Xu et al., assessed the effect of in utero exposure to pets (woman having a pet during pregnancy) on the risk of hypertension from air pollutants later in life [16•]. Children not exposed to home pets had a significantly stronger hypertensive response to air pollution. For example, the highest odds ratios per 30.6 μg/m^3^ increase in PM_10_ (inhalable particles, with diameters that are generally 10 μm and smaller) were 1.79 (95% *CI*: 1.29–2.50) in children without current pet exposure compared to 1.24 (95% *CI*: 0.85–1.82) in children with current pet exposure. Furthermore, the increases in mean diastolic BP per 46.3 μg/m^3^ increase in O_3_ (ozone) were 0.60 mmHg (95% *CI*: 0.21–0.48 mmHg) in children without pet exposure in utero and 0.34 mmHg (95% *CI*: 0.21–0.48 mmHg) in those with in utero pet exposure. Thus, in utero exposure to a domestic animal mitigated the negative impact of air pollutants on the risk of developing hypertension later in life [16•].

Based on the available observational and interventional studies, it is not possible to conclusively determine whether having pets reduces BP and the risk of hypertension in the general population. The elderly and children who have a pet appear to benefit most in reducing high BP and the risk of hypertension. Importantly, having pets can alleviate the prohypertensive properties of environmental exposure to tobacco smoke or air pollution.

## Pet Ownership, BP Control and CVD Risk in Hypertensive Patients

**Fig. 1:**
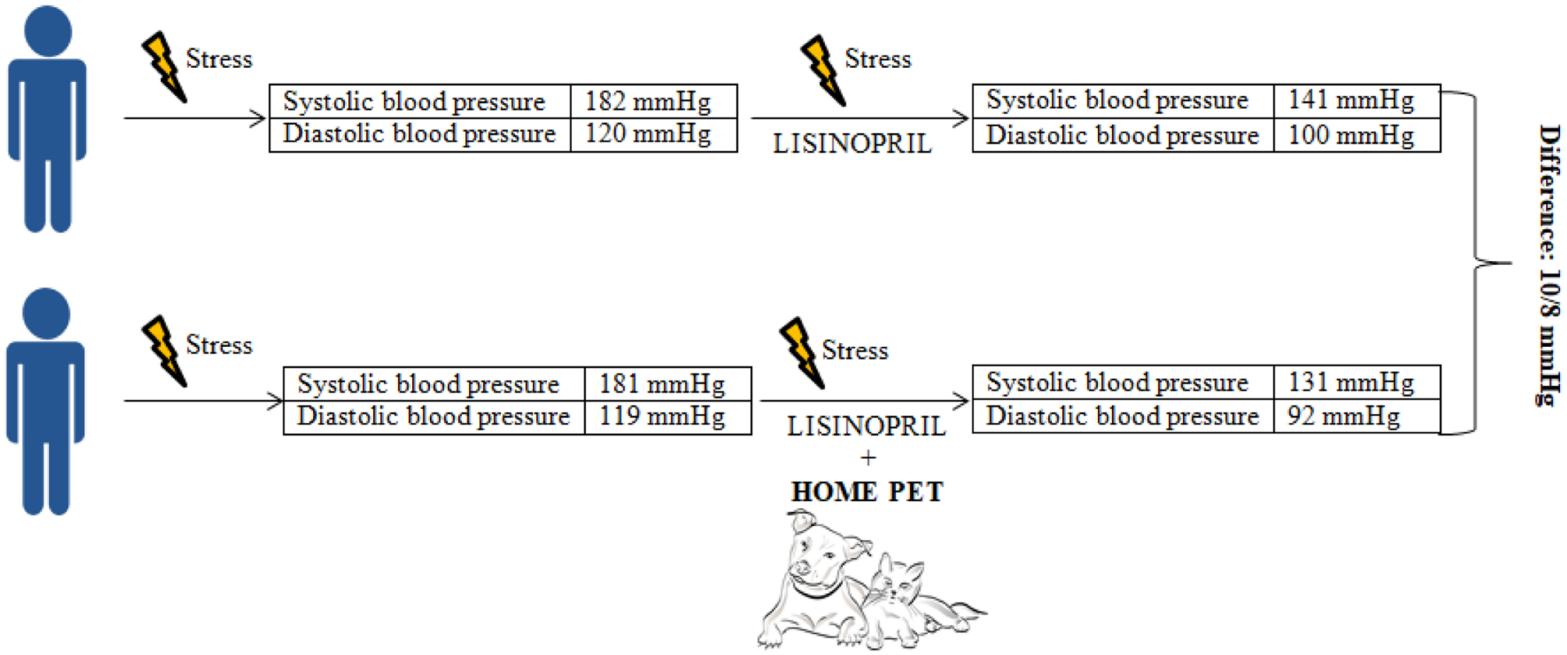
Effect of a home pet on reducing BP in persons with stress-induced BP increases. Based on [17]

A study by Allen et al. analyzed the impact of having a pet on BP, HR, and plasma renin activity in 48 patients with hypertension who were exposed to stress (mathematical task) [17]. Participants were divided into two groups. The first received lisinopril (20 mg/day), and the second was given a pet, in addition to lisinopril. Having a pet increased the antihypertensive effect of lisinopril and decreased the HR and plasma renin activity responses to stress (Fig. 1) [17]. This interventional study provided evidence that having a pet may contribute to better BP control in patients with hypertension.

Similar results were obtained by Friedmann et al. in an observational study of the effects of owning a dog or cat on BP in 32 people aged 50–83 with SBP in 120–150 mmHg and DBP in 80–100 mmHg or SBP < 150 mmHg and DBP < 100 mmHg with antihypertensive medications [18]. Of these, 21 participants had a dog, 8 had a cat, and 3 had both a dog and a cat. Two thirds had hypertension. Changes in BP were assessed using ambulatory BP monitoring (ABPM). After accounting for confounding factors, having a dog was associated with decreases in SBP (− 2.5 mmHg) and DBP (− 1 mmHg), and having a cat was associated with a decrease in DBP (− 1.5 mmHg) and an increase in SBP (+ 4.5 mmHg) [18].

A subgroup study of the Second Australian National Blood Pressure (ANBP2) Study assessed the effects of having pets on the risk of all cause- and CVD-related death in 4039 participants aged 65–84 years with untreated hypertension (≥ 160 mmHg SBP and/or ≥ 90 mmHg DBP) at randomization [19]. Over an 11 year observation period, 958 participants died and 499 deaths were related to CVD events. Having a pet (versus never own a pet) was associated with reduced risk of death from any cause (previous pet owner: HR = 0.76; 95% *CI*: 0.63–0.91; current pet owner: HR = 0.72; 95% *CI*: 0.60–0.87) and from CVD causes (previous pet owner: HR = 0.70; 95% *CI*: 0.55–0.89; current pet owner: HR = 0.60; 95% *CI*: 0.46–0.77) [19]. Thus, having pets may improve BP control and prognosis in patients with hypertension.

## Pet Ownership and Risk of Stroke, Myocardial Infarction, Coronary Artery Disease and Heart Failure

There is no published evidence that having a pet alters the risk of stroke or myocardial infarction [3••, 9••]. However, dog ownership has been associated with lower mortality risk in patients with a history of stroke (HR = 0.82; 95% *CI*: 0.78–0.86) or myocardial infarction (HR = 0.79; 95% *CI*: 0.75–0.83) [20••]. Furthermore, the Cardiac Arrhythmia Suppression Trial (CAST) showed that possession of home pets was associated with lower mortality risk in the first year after a myocardial infarction [21]. Beneficial effects of pet ownership on the prognosis of patients after major CVD events have also been shown in other studies [22]. Furthermore, the results of the study by Xie et al. indicate that having a dog likely reduced the risk of coronary artery disease [23]. However, another large population study did not detect an effect of home pets on the risks of coronary artery disease or heart failure (HF) [9••].

Together, results of these studies suggest that having pets does not reduce the risk of stroke, and HF overall, but may reduce the risk of coronary artery disease. Moreover, having pets does reduce the risk of death from stroke and myocardial infarction.

## Possible Mechanisms Mediating the Cardiovascular Benefits of Pet Ownership

The beneficial effects of pet ownership on the cardiovascular (CV) system have been attributed to increased vagal tone and reduced sympathetic drive. A study by Matook et al. found that activity of the parasympathetic nervous system was greater in healthy people who walked their dogs compared to those who walked without a dog. Furthermore, staying with the dog at home resulted in enhanced activity of the parasympathetic nervous system [24]. A study by Cole assessed the effect of a 12-min visit by a volunteer with a dog on CV function in 76 patients with HF [25]. Patients were divided into three groups. The first was visited by a volunteer with a dog, the second was visited by a volunteer alone, and the third was visited by hospital staff (control group). The presence of a dog led to improvements in CV indicators such as pulmonary artery pressure, wedge pressure, and blood epinephrine levels, as well as decreases in anxiety levels in HF patients [25]. A recently published pilot study by Ortmeyer and Katzel assessed the impact of pets on HR variability (HRV) [26]. Results from the pilot study support the hypothesis that spending time in the presence of a companion dog increases caregivers’ HRV throughout the day and suggest that proximity to a dog may contribute to overall improvement in 24-h HRV and cardiac health in dog caregivers. It is worth emphasizing that the influence of home pets on the activity of the autonomic nervous system may depend on whether the animal is known or foreign (the person has never had contact with this animal before the study) to the human. A study by Kingwell et al. found that a friendly but unfamiliar dog does not influence BP or HR either at rest or during mild mental stress. The cardiac autonomic profile was improved in dog owners in the presence of a pet dog and in non-dog owners in the absence of a dog. [27].

Among the potential mechanisms of the health promoting impact of pet ownership are increased physical activity, dietary improvement, and smoking cessation. The study of Ogechi et al. analyzed data from 3964 persons without serious CVD from the NHANES III survey, of whom 34.6% declared having a pet [28]. Having a pet was associated with significant differences in the lifestyle of the respondents. Pet owners were more often younger, married, white, and drank alcohol and smoked cigarettes less often. These observations were confirmed in the study by Maugeri et al. which showed a statistically significant relationship between having a pet and greater physical activity, a healthier diet and not smoking [12]. A study by Powell et al. also found that having a dog was associated with more daily steps and increased physical activity [29•]. A review of the literature by Christian et al. also showed a beneficial effect of having pets on increasing the physical activity of the owners [30].

**Fig. 2:**
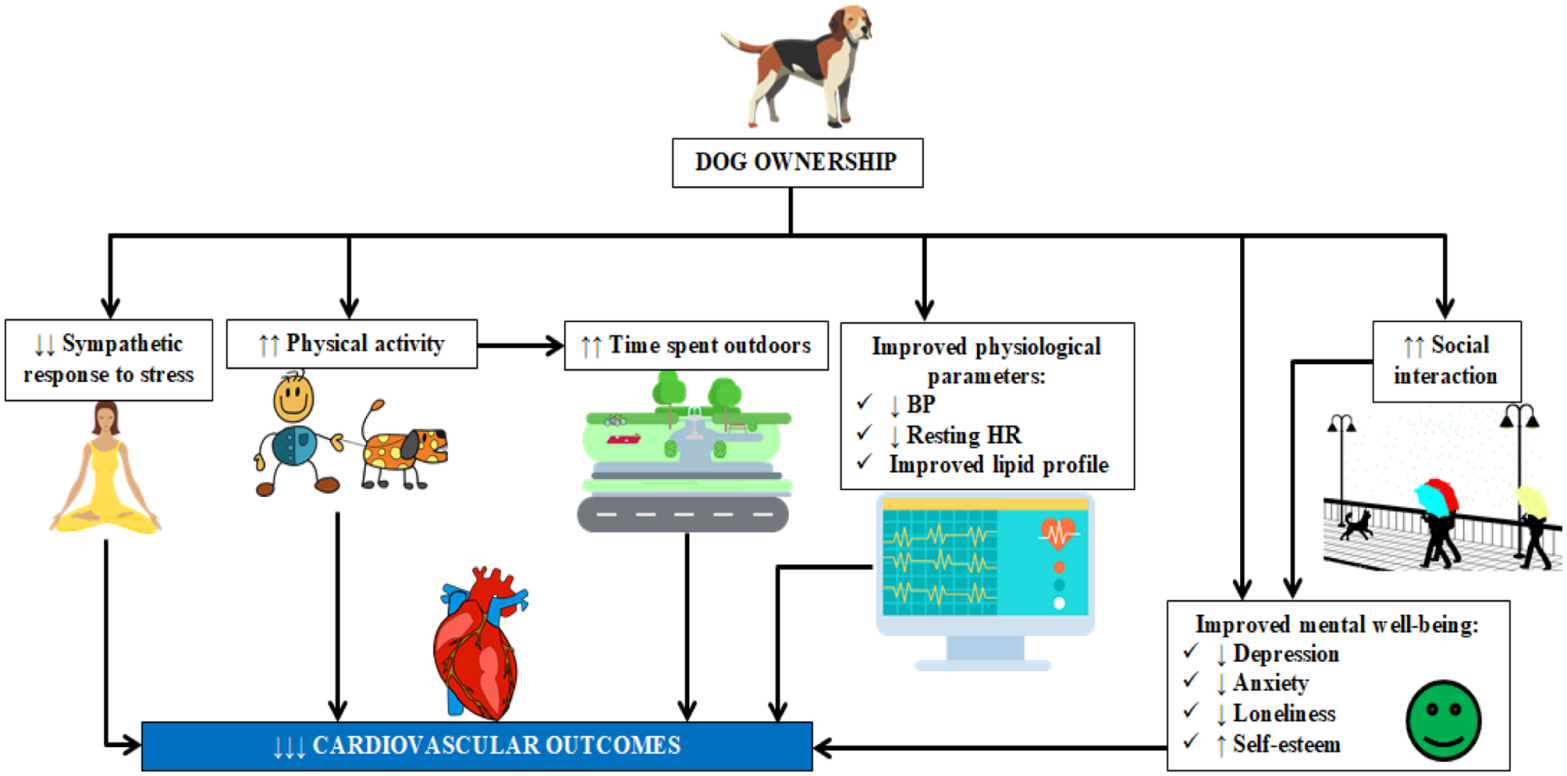
Potential mechanisms for the cardiovascular benefit of dog ownership. Based on [1, 32]

Dog ownership appears to have the important potential health effects, as summarized in Fig. 2 [1, 32]. However, as presented in this study and in the review by Arhant-Sudhir et al. many potential factors affecting the CV health of pet owners remain controversial (Table 3) [31].

**Table 1:**
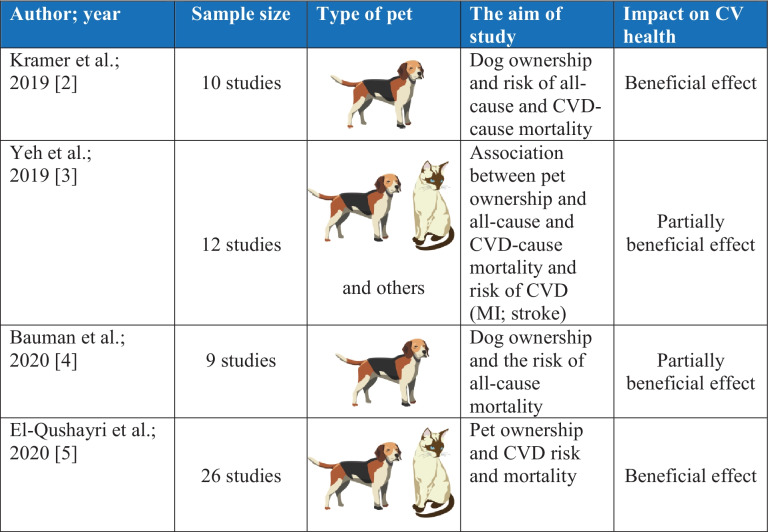
Summary of the results of meta-analyzes assessing the impact of having pets on the risk of death. *CV*, cardiovascular; *CVD*, cardiovascular disease; *MI*, myocardial infarction

## Factors Limiting the Interpretation of Research

Some confounding factors may explain the discrepancies in the results of studies analyzing the CV effects of pet ownership. Pet owners tend to be wealthier, better educated, and married [1]. Furthermore, the link between dog ownership and good health may not be causal, as adults with excellent physical health are more likely to adopt a dog than those who are too sick or too weak to have a pet [1].

## Clinical Recommendations and Conclusions

Pet ownership appears to have a positive effect on the CV system. The discrepancies and inconclusive results of some available studies may be caused by confounding factors and differences in research methodology. Furthermore, some studies were observational and others interventional, limiting the ability to compare their results and draw definitive conclusions. In their clinical recommendations, the American Heart Association (AHA) points to pet ownership as a way to mitigate CVD risk. They conclude that pet ownership, especially ownership of a dog, may be associated with decreased CVD risk (Table 4). However, they caution that CVD risk reduction should not be the primary motivation behind pet adoption, rescue, or purchase [33]. Owning a pet should be based on sympathy and responsibility (caring for and treating the animal well) to the pet, while possible health benefits for the owner are only an added value and not an end in itself.

**Table 2:**
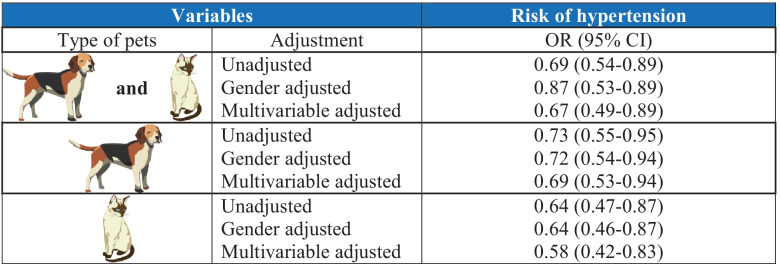
The risk of hypertension depending on the type of pet owned. Based on [9••]. *OR*, odds ratio; *95% CI*, 95% confidence interval

**Table 3:** Beneficial effects of pet ownership on cardiovascular risk. *SBP*, systolic blood pressure; *MAP*, mean arterial pressure; *TG*, triglycerides. Based on [31]

Cardiovascular risk factor	Beneficial effect of pet ownership	Conflicting data
Sedentary lifestyle	Increases regular exercise, frequency of walking, especially for leisure	Yes
Arterial hypertension	Lower SBP, pulse pressure, MAP	Yes
Stress	Lower BP response to mental stress	Yes
Hyperlipidemia	Lower plasma TG and cholesterol	Yes
Post myocardial infarction arrhythmias or re-infarction	Improved survival in the year following myocardial infarction	Yes
Depression	Fewer physician visits, less depression	Yes

**Table 4:** Clinical recommendations of the American Heart Association for having pets as a method of cardiovascular prevention. Based on [33]

Recommendation	Class	Level
Pet ownership, particularly dog ownership, may be reasonable for reduction in CVD risk	IIb	B
Pet adoption, rescue, or purchase should not be done for the primary purpose of reducing CVD risk	III	C

Legend: Class IIb. may/might be reasonable; III, should not be performed/administered/other

Level B, recommendations are based on limited or inconsistent scientific evidence; C, recommendations are based primarily on consensus and expert opinion

Owning pets is important on a global scale. Many new studies have been published since the AHA position paper on the CV effects of pet ownership in 2013, but the relationship is still not fully understood. Generally, having pets appears to have a positive effect on the CV system, but a number of inconsistencies and contradictory results are caused by confounding factors, differences in research methodology, and the fact that some studies were observational and some interventional, which makes it difficult to compare results and draw conclusions. Probably, people who like pets get more health benefits from having pets. Prospective and interventional studies should be carried out to improve knowledge of the effects of pet ownership on the CV system and CVD risk. Existing evidence of the CVD benefits of pet ownership is sufficiently weak that the sole purpose of having pets should not be to prevent CVD.
